# Outcome of Tocilizumab Treatment in Febrile Neutropenic Children with Severe Sepsis/Septic Shock in a Single-Center Retrospective Case Series

**DOI:** 10.3390/cancers16081512

**Published:** 2024-04-16

**Authors:** Shih-Hsiang Chen, Tsung-Yen Chang, Yi-Lun Wang, En-Pei Lee, Jainn-Jim Lin, Yi-Wen Hsiao, Tang-Her Jaing, Chao-Ping Yang, Iou-Jih Hung

**Affiliations:** 1 Division of Pediatric Hematology-Oncology, College of Medicine, Chang Gung Memorial Hospital, Chang Gung University, Taoyuan 333, Taiwan; gisborne@cgmh.org.tw (T.-Y.C.); mp2601@cgmh.org.tw (Y.-L.W.); jaing001@cgmh.org.tw (T.-H.J.); ilvlct@cgmh.org.tw (C.-P.Y.); mat423@cgmh.org.tw (I.-J.H.); 2 Division of Pediatric Critical Care Medicine, College of Medicine, Chang Gung Memorial Hospital, Chang Gung University, Taoyuan 333, Taiwan; lnp1511@cgmh.org.tw (E.-P.L.); lin0227@adm.cgmh.org.tw (J.-J.L.); 3 Department of Nursing, College of Medicine, Chang Gung Memorial Hospital, Chang Gung University, Taoyuan 333, Taiwan; apple80168@cgmh.org.tw

**Keywords:** children, febrile neutropenia, severe sepsis, septic shock, tocilizumab

## Abstract

**Simple Summary:**

Febrile neutropenia is common in children with hemato-oncological disease. It could be fatal, especially when severe sepsis/septic shock occurs. The aim of our retrospective study was to investigate the efficacy of tocilizumab on the outcome of febrile neutropenic children with severe sepsis/septic shock. We found that Il-6 blockade with tocilizumab is a potential therapeutic strategy for severe sepsis/septic shock in seven children with febrile neutropenia. Two of four (50%) patients receiving tocilizumab therapy needed pediatric intensive care unit (PICU) admission, whereas all three (100%) patients without tocilizumab therapy were admitted to the PICU. None of the four (0%) patients receiving tocilizumab therapy died of an episode of severe sepsis/septic shock. However, two of three (67%) patients without tocilizumab therapy died of rapid progression of multiple organ failure after the development of severe sepsis/septic shock. Further research to investigate the efficacy and long-term safety in a larger number of patients with a longer follow-up is needed.

**Abstract:**

Purpose: To assess the efficacy of an IL-6 blockade with tocilizumab on treatment outcome of severe sepsis/septic shock in children with febrile neutropenia. Methods: We performed a retrospective study of febrile neutropenic patients younger than 18 years old who developed severe sepsis/septic shock at a single medical center between November 2022 and October 2023. Results: Seven patients with febrile neutropenia complicated with severe sepsis/septic shock were identified. Four of seven patients received tocilizumab in addition to standard of care. The median IL-6 level before administration of tocilizumab was 14,147 pg/mL (range: 672–30,509 pg/mL). All four patients successfully recovered from severe sepsis/septic shock. Three of seven patients received standard of care without tocilizumab. IL-6 levels were checked intwo2 patients, with a median of 1514.5 (range: 838–2191). Only one of three (33%) patients without tocilizumab therapy made a full recovery from severe sepsis/septic shock. The mortality rate was higher in patients without tocilizumab therapy compared to patients with tocilizumab therapy (67% vs. 0%). Conclusions: Administration of tocilizumab reduced mortality of severe sepsis/septic shock in children with febrile neutropenia. However, it warrants confirmation with a larger number of patients and a longer follow-up.

## 1. Introduction

Febrile neutropenia is common in children with hemato-oncological disease, especially after receiving myelosuppressive or immunosuppressive therapy. The mortality rate of untreated or inappropriate treatment of febrile neutropenia was 2–21% [1]. However, mortality could be up to 41–46% when children with hemato-oncological diseases develop severe sepsis/septic shock [2]. Therefore, not only early administration of broad-spectrum antibiotics and/or antifungal agents but also positive developments in supportive therapies for severe sepsis/septic shock are believed to improve the outcome of febrile neutropenia in children with hemato-oncological disease.

Clinical practice guidelines for febrile neutropenia in children with hemato-oncological diseases were first published in 2012 and updated in 2023 [3]. In high-risk patients, it was recommended to use monotherapy with an antipseudomonal β-lactam, a fourth-generation cephalosporin, or a carbapenem as empiric antibacterial therapy. The addition of a second anti-Gram-negative agent or a glycopeptide was reserved for patients who were clinically unstable or when a resistant infection was suspected. The Surviving Sepsis Campaign developed an evidence-based guideline for the management of septic shock and sepsis-associated organ dysfunction in children [4]. The guideline recommended starting antimicrobial therapy as soon as possible, within 1 h of recognition of children with septic shock. In children with sepsis-associated organ dysfunction (i.e., severe sepsis), it was recommended to start antimicrobial therapy as soon as possible after appropriate evaluation, within 3 h of recognition. Fluid therapy was suggested to be administered in up to 40–60 mL/kg in bolus fluid (10–20 mL/kg per bolus) over the first hour if intensive care was available. Epinephrine and norepinephrine were considered first-line vasoactive agents for the treatment of septic shock. Dopamine might still be used if epinephrine or norepinephrine was not readily available.

Although guidelines for the management of severe sepsis/septic shock are constantly developed [5,6], severe sepsis/septic shock in children with hemato-oncological diseases is frequently devastating and unresponsive to recommended therapies [7]. It was reported that a high IL-6 level was observed both in children with cytokine release syndrome (CRS) after chimeric antigen receptor (CAR) T-cell therapy and in children with sepsis [8]. Tocilizumab is a humanized anti-human IL-6 receptor monoclonal antibody approved for the treatment of rheumatoid arthritis, systemic juvenile idiopathic arthritis, polyarticular juvenile rheumatoid arthritis, giant cell arteritis, systemic sclerosis-associated interstitial lung disease, CRS, and coronavirus disease 2019 (COVID-19). In COVID-19 critical cases, a remarkable rise in the IL-6 level in blood was associated with sepsis or septic shock [9]. A meta-analysis of clinical trials of patients hospitalized for COVID-19 demonstrates that the administration of tocilizumab was associated with lower 28-day all-cause mortality compared to standard care or placebo [10].

Based on the successful experience from COVID-19 critical cases, it is reasonably hypothesized that IL-6 is able to restore hemodynamic stability in children with febrile neutropenia who develop severe sepsis/septic shock. Previously, one case series recruiting four adult patients with hematological diseases concluded that tocilizumab may help reduce the risk of organ injury following sepsis [11]. However, the efficacy of tocilizumab on the treatment of severe sepsis/septic shock among pediatric patients with febrile neutropenia remains unclear. Here, we present our experience of using tocilizumab in pediatric patients with febrile neutropenia who developed severe sepsis/septic shock. The aim of this study was to assess the efficacy of an IL-6 blockade with tocilizumab on the treatment outcome of sepsis/septic shock in children with febrile neutropenia.

## 2. Methods

### 2.1. Study Design

This study followed a retrospective observational cohort design. We investigated, by manual chart review, consecutive patients in the Pediatric Hematology/Oncology ward in Chang Gung Memorial Hospital, Taoyuan, Taiwan, who received myelosuppressive or immunosuppressive therapy for underlying hemato-oncological diseases between November 2022 and October 2023. Patients with febrile neutropenia and severe sepsis/septic shock were identified based on the following criteria. Fever was defined as a single body temperature >38.5 °C or ≥2 episodes of temperature >38.0 °C within a 6 h period. Neutropenia was defined as absolute neutrophil count (ANC) <0.5 × 10^9^/L or ANC 1 × 10^9^/L with a predicted decrease to <0.5 × 10^9^/L in the next 48 h. The diagnosis of severe sepsis/septic shock was based on the 2005 International Pediatric Sepsis Consensus Conference [12]. Briefly, severe sepsis was defined as sepsis plus one of the following: cardiovascular organ dysfunction or acute respiratory distress syndrome or ≥2 other organ dysfunctions. Septic shock was defined as sepsis plus cardiovascular organ dysfunction. Patients who received blinatumomab immunotherapy or CAR-T therapy were excluded.

### 2.2. Treatment of Febrile Neutropenia with Sepsis/Septic Shock

All children with febrile neutropenia initially received standard evaluation and treatment in our pediatric hematology/oncology ward as previously reported [13,14]. When hypotension developed, rapid fluid resuscitation, given as a 10–20 mL/kg bolus of isotonic crystalloid (usually 0.9% normal saline), was infused and repeated twice if necessary. The strategies to adjust empirical antibiotic therapy and antifungal therapy were reported previously [13]. Inotropic agents (mainly dopamine) were used rapidly if blood pressure did not respond to fluid resuscitation well. When respiratory distress developed, oxygenation therapy with a high-flow nasal cannula, simple face mask, or non-rebreathing mask was applied. If persistent hypotension, persistent desaturation, and/or the development of consciousness disturbance were observed, the patients were transferred to the pediatric intensive care unit (PICU). At the PICU, blood biochemistries were checked to evaluate organ dysfunction, including blood urea nitrogen (BUN), creatinine, aspartate transaminase (AST), alanine transaminase (ALT), creatine kinase (CK), creatine kinase MB (CK-MB), troponin-I (TnI), and N terminal pro B-type natriuretic peptide (NT-proBNP). Epinephrine and/or norepinephrine were substituted for dopamine to treat septic shock. Nasal continuous positive airway pressure therapy, nasal intermittent positive pressure ventilation therapy, or intubation with mechanical ventilation therapy were applied, depending on the respiratory status of the patients.

### 2.3. Administration of Tocilizumab and Measurement of Serum IL-6 Level

Measurement of serum IL-6 level and administration of tocilizumab were routinely suggested, but not mandatory, when severe sepsis/septic shock was suspected or diagnosed. Administration of tocilizumab for conditions other than rheumatoid arthritis or juvenile idiopathic arthritis and laboratory tests for IL-6 level were both not covered by National Health Insurance in Taiwan. The parents could make the decision to refuse one or both after discussing with the treated physicians. Therefore, blood samples were collected to test IL-6 level, and tocilizumab was administered only when we obtained informed consent for the self-pay agreement from the parents. Tocilizumab was administered intravenously as a single dose at 8 mg/kg for weight ≥ 30 kg or 12 mg/kg for weight < 30 kg.

### 2.4. Statistical Analysis

Fisher’s exact test and Wilcoxon’s rank-sum test were used as appropriate to make comparisons between groups. Statistical analyses were performed using R version 2.9.1 for Windows (www.r-project.org). In all analyses, *p*-values were two tailed and considered statistically significant at <0.05.

## 3. Results

### 3.1. Clinical and Laboratory Characteristics of the Patients

Between November 2022 and October 2023, 123 patients were admitted to our Pediatric Hematology/Oncology ward for myelosuppressive or immunosuppressive therapy. The total number of admissions was 688. There were 118 episodes of febrile neutropenia in 59 patients. Documented or clinical sepsis was diagnosed in 17 episodes of febrile neutropenia in 14 patients. Among these fourteen patients, seven complicated with severe sepsis/septic shock were identified (Table 1). There were three boys and four girls. The median age of developing severe sepsis/septic shock was 13.4 years (range: 6.7–17.3 years). The diagnoses of underlying diseases included severe aplastic anemia (SAA) in three, acute myeloid leukemia (AML) in two, acute lymphoblastic leukemia (ALL) in one, and malignant germ cell tumor (MaGCT) in one. All patients had severe sepsis. Six of seven patients had profound hypotension requiring inotropic agents, which fulfilled the definition of septic shock.

Four of seven patients received tocilizumab therapy in addition to standard of care. The median level of IL-6 before administration of tocilizumab was 14,147 pg/mL (range: 672–30,509 pg/mL). Concomitant C-reactive protein (CRP), procalcitonin (PCT), and ferritin levels were checked in three, two, and two patients, respectively. The median levels of CRP, PCT, and ferritin were 178 mg/L, 47.7 ng/mL, and 10,890.5 ng/mL, respectively. Three of seven patients did not receive tocilizumab therapy. Two had IL-6 levels measured on the first day of septic shock. The median level of IL-6 was 1514.5 pg/mL (range: 838–2191 pg/mL). CRP, PCT, and ferritin levels were measured in all three patients. The median levels of CRP, PCT, and ferritin were 136.01 mg/L, 8.87 ng/mL, and 1567 ng/mL, respectively. Although the median levels of inflammatory markers were higher in patients receiving tocilizumab therapy, they were not statistically significant (Table 2).

Tocilizumab was administered within 10 h after the onset of febrile neutropenia in three of four patients receiving tocilizumab therapy. The remaining patient received tocilizumab 5 days after the onset of febrile neutropenia. The median duration of neutropenia before the onset of febrile neutropenia with severe sepsis/septic shock was 3 days (range: 2–7 days) in patients receiving tocilizumab therapy, which was statistically significantly shorter than patients without tocilizumab therapy (median: 20 days; range: 9–47 days; *p* = 0.049).

### 3.2. Treatment Outcome of the Patients

Patients without tocilizumab tended to need PICU admission. Two of four (50%) patients receiving tocilizumab therapy needed PICU admission, whereas all three (100%) patients without tocilizumab therapy were admitted to the PICU. The mortality rate tended to be higher in patients without tocilizumab therapy. None of the four (0%) patients receiving tocilizumab therapy died of an episode of severe sepsis/septic shock. However, two of three (67%) patients without tocilizumab therapy died of rapid progression of multiple organ failure after the development of severe sepsis/septic shock. Although there were trends for a higher PICU admission and mortality in patients without tocilizumab therapy, it did not reach statistical significance.

### 3.3. Detailed Information of the Patients

Patient 1 was diagnosed with relapsed AML after a transplant. She underwent salvage chemotherapy with mitoxantrone and etoposide. She started prophylactic antibiotic therapy with intravenous vancomycin and oral levofloxacin after completing the 5-day chemotherapy. She developed febrile neutropenia and a cough on the 13th day post-chemotherapy. Antibiotic therapy was modified as teicoplanin and ceftazidime empirically. One day later, she had a rapid progression of dyspnea requiring a simple face mask at flow rates of 6 L/min. Neurologic symptoms, including visual hallucination and delirium with a drop in the Glasgow coma scale (GCS) of 3 points, were subsequently observed. She was transferred to the PICU for further evaluation and management of her unconsciousness and respiratory distress. The computed tomography of the brain showed the absence of an intracranial hemorrhage. The cerebrospinal fluid (CSF) analysis did not show pleocytosis, and the culture did not yield any pathogen. A throat swab sample was collected and tested with the FilmArray Respiratory Panel. The result revealed the exclusive presence of parainfluenza virus–3. Parainfluenza virus–3 was also isolated from the throat swab sample. Despite aggressive supportive care and adjustment of antimicrobial therapy, her fever, respiratory distress, and neurological symptoms did not improve much. One dose of tocilizumab (8 mg/kg) was administered intravenously on the 5th day of PICU admission. Her fever resolved, her GCS was improved, and her oxygen demand was reduced within the next 24 h. Her serum IL-6 level before administration of tocilizumab was 1200 pg/mL (normal < 7).

Patient 2 was diagnosed with acquired SAA. He underwent frontline rabbit antithymocyte globulin (ATG) and cyclosporine-based immunosuppressive therapy because he did not have a matched sibling donor. He developed febrile neutropenia and bilateral knee joint pain on the 9th day post-exposure to rabbit ATG. Teicoplanin and ceftazidime were administered empirically. However, he had remittent fever, tachycardia, and tachypnea in the following 6 h. Hypotension developed subsequently. Fluid resuscitation and inotropic support with dopamine were administered. Meropenem was substituted for ceftazidime empirically. Blood biochemical tests showed normal renal and hepatic functions. One dose of tocilizumab (12 mg/kg) was administered intravenously. His fever resolved within 4 h. His blood pressure also increased gradually, and dopamine was discontinued 3 days later. His serum IL-6 level before administration of tocilizumab was 672 pg/mL (normal < 7).

Patient 3 was diagnosed with de novo AML. She received chemotherapy with fludarabine, cytarabine, and granulocyte colony-stimulating factor (FLAG regimen). She started prophylactic antibiotic therapy with intravenous vancomycin and oral levofloxacin after completing the 5-day chemotherapy. Febrile neutropenia occurred on the 12th day post-chemotherapy. Her fever reached 40 °C, followed by agitation, oliguria, and hypotension. Blood biochemical tests showed an elevation of BUN (21.2 mg/dL, normal 5–20), creatinine (1.42 mg/dL, normal 0.2–1.0), AST (207 U/L, normal 13–40), and ALT (391 U/L, normal 7–40), suggesting the ongoing multiple organ failure. In addition to fluid resuscitation and inotropic support with dopamine, antibiotic therapy was switched to teicoplanin and meropenem. One dose of tocilizumab (8 mg/kg) was administered intravenously. Her fever resolved in the next 6 h. Her blood pressure became normalized, and dopamine was discontinued 3 days later. Her serum IL-6 level before administration of tocilizumab was 27,094 pg/mL (normal < 7). The blood culture yielded *Brevundimonas* species.

Patient 4 was diagnosed with relapsed MaGCT. He received salvage chemotherapy with paclitaxel and gemcitabine administered on days 1, 8, and 15. He developed febrile neutropenia and respiratory distress with desaturation on the 18th day post-chemotherapy. After initial fluid resuscitation, he was transferred to the PICU rapidly. Blood biochemical tests showed an elevation of BUN (41.3 mg/dL, normal 5–20), creatinine (4.24 mg/dL, normal 0.2–1.0), AST (206 U/L, normal 13–40), ALT (321 U/L, normal 7–40), CK (2924 U/L, normal 56–224), CK-MB (76.2 ng/mL, normal < 5.1), TnI (1.783 ng/mL, normal < 0.026), and NT-proBNP (1.783 pg/mL, normal < 125). He needed a non-rebreathing mask at flow rates of 15 L/min and two inotropic agents (epinephrine and norepinephrine) to maintain his hemodynamic stability. Teicoplanin and meropenem were administered empirically. One dose of tocilizumab (8 mg/kg) was administered intravenously. His fever resolved in the next 12 h. Both inotropic agents could be discontinued 5 days later. His hepatic, renal, and cardiac markers recovered in 10 days. He did not receive any further intensive treatment (e.g., intubation and dialysis). His serum IL-6 level before administration of tocilizumab was 30,509 pg/mL (normal < 7). The blood culture yielded *Aeromonas* species.

Patient 5 was diagnosed with TCF3:PBX1-positive ALL and she had persistent minimal residual disease after chemotherapy. She underwent peripheral blood stem cell transplantation from a human leukocyte antigen-matched sibling donor. Unfortunately, a relapse of ALL was confirmed on the 79th day after the transplant. She received three courses of salvage chemoimmunotherapy with a modified hyper-CVAD and rituximab regimen. She developed pancytopenia followed by skin rashes, hypertransaminasemia, and hyperbilirubinemia, suggesting grade 2 acute graft-versus-host disease. Cyclosporine was administered accordingly. She had two episodes of febrile neutropenia without documented or clinical sepsis. Antimicrobial therapy had been escalated to teicoplanin and meropenem. Although no pathogens were isolated, antimicrobial therapy continued without modification because severe neutropenia did not resolve. The third episode of febrile neutropenia occurred on the 47th day of neutropenia. She had a progressive onset of dyspnea and hypotension in the following 2 days. She was transferred to the PICU rapidly. Unfortunately, she did not respond to aggressive intensive therapy and was dead 2 days later. The indwelling central venous catheter tip culture yielded *Staphylococcus pettenkoferi*.

Patient 6 was diagnosed with hepatitis-associated SAA. She underwent frontline rabbit ATG and cyclosporine-based immunosuppressive therapy. She developed a first episode of febrile neutropenia caused by carbapenem-resistant *Klebsiella pneumoniae* on the 7th day post-exposure to rabbit ATG. It was successfully treated by antimicrobial therapy with meropenem and amikacin. She developed a second episode of febrile neutropenia with coughing 10 days later. Radiological images showed new infiltrations in the right lower lung, suggesting infection. She had progressive onset of hypotension, respiratory distress, and desaturation within 2 days. She was transferred to the PICU rapidly. Unfortunately, she did not respond to aggressive intensive therapy and was dead 2 days later. The blood culture yielded *Ralstonia mannitolilytica* and *Stenotrophomonas maltophilia*.

Patient 7 was diagnosed with acquired SAA. He underwent frontline rabbit ATG and cyclosporine-based immunosuppressive therapy. He developed febrile neutropenia on the 10th day post-exposure to rabbit ATG. Teicoplanin and ceftazidime were administered empirically. However, he developed hypotension subsequently. His blood pressure did not improve after fluid resuscitation. He was transferred to the PICU rapidly. Blood biochemical tests showed normal hepatic, renal, and cardiac markers. Inotropic support with epinephrine was administered. Meropenem was substituted for ceftazidime empirically. His general condition improved gradually, and epinephrine was discontinued 3 days later. He recovered from septic shock successfully. The blood culture was negative.

## 4. Discussion

Febrile neutropenia is commonly encountered in patients receiving myelosuppressive or immunosuppressive therapy to treat underlying hemato-oncological disease. Febrile neutropenia can lead to severe sepsis/septic shock, which continues to be the leading cause of intensive care unit admission and mortality in patients with hemato-oncological disease after medical treatment [15,16].

CRS is a systemic inflammatory response, and unfettered cytokine release can occur in many conditions, including infections. It has been reported in patients infected by cytomegalovirus, Epstein–Barr virus, SARS-CoV, H5N1 influenza virus, and, most recently, SARS-CoV-2 [17]. It has been reported that the serum level of several cytokines is frequently raised in sepsis [18]. Recently, elevated serum IL-6 was found to be associated with respiratory failure, acute respiratory distress syndrome, and poor clinical outcomes in patients with COVID-19 [19].

Cytokines also play a crucial role in children with febrile neutropenia. We previously investigated IL-8 and CRP levels in 30 children with febrile neutropenia after allogeneic hematopoietic stem cell transplantation. Although IL-8 was positively correlated to CRP in febrile neutropenia, IL-8 level was not associated with the presence and development of clinical infection [14]. In 35 children with febrile neutropenia, de Araujo et al. found that the concentrations of IL-6, IL-8, and IL-10 on day 1 of fever were significantly higher in septic patients compared to nonseptic patients [20]. Consistently, IL-6 was one of the powerful discriminators to detect infectious etiology in another study analyzing 110 episodes of febrile neutropenia in children [21]. IL-6 level on day 4 of febrile neutropenia was significantly higher in the episodes with a microbiologically documented infection.

A recent study demonstrated that serum IL-6 levels could distinguish between sepsis and septic shock. Patients with high serum IL-6 levels (≥348.9 pg/mL) significantly proceeded to septic shock and were associated with a higher rate of 28-day mortality [22]. Furthermore, the elevation of serum IL-6 levels in children admitted to the PICU showed a linear relationship with an increased risk of new or progressive multiple organ failure incidence [23]. Serum IL-6 levels rapidly increased in patients with sepsis within 2 h of infection onset [24]. Thao et al. later showed that an ≥86% reduction in IL-6 level within 24 h after admission to the intensive care unit was significantly associated with a significant overall survival advantage in patients with sepsis and septic shock [25]. Therefore, it is reasonable that IL-6 would be the targeted cytokine in severe sepsis/septic shock in children with febrile neutropenia. Early blockage of IL-6 by tocilizumab in children with severe sepsis/septic shock could reduce the risk of the development of multiple organ failure and increase the survival outcome. As reported in patient 3 and case 4, tocilizumab was administered rapidly when severe sepsis/septic shock was diagnosed. The circulatory failure was corrected successfully, and even profound organ dysfunction had occurred in patient 4.

Although tocilizumab is very effective in treating CRS after CAR-T therapy, it has little effect in most cases of immune effector cell-associated neurotoxicity syndrome (ICANS) [26]. In an animal model, systemic administration of tocilizumab did not result in good penetration into the brain [27]. Furthermore, it was reported that serum IL-6 levels increased after the administration of tocilizumab due to the blockage of the receptor in the peripheral tissue. Therefore, the occurrence of severe ICANS was increased, whereas the development of CRS was reduced [26,28]. Muccioli et al. reported that neuropsychiatric symptoms in a patient with COVID-19 were promptly resolved after treatment with tocilizumab [29]. The breakdown of the blood–brain barrier (BBB) caused by SARS-CoV-2 could help tocilizumab pass freely into the brain and improve its therapeutic effect on neurological symptoms [30]. Severe sepsis can induce dysfunction of the BBB and increase BBB permeability by the disruption of tight junction proteins [31]. We speculated that parainfluenza virus-3 infection led to the disruption of BBB in patient 1. As a result, tocilizumab could penetrate into the brain easily, and GCS improved rapidly after tocilizumab therapy.

The median IL-6 level was higher in patients receiving tocilizumab therapy than those without tocilizumab therapy. However, two (patients 1 and 2) of four patients with tocilizumab had IL-6 levels similar to two (patients 5 and 6) of three patients without tocilizumab. We speculated that the timing of tocilizumab administration might play a role in the successful recovery. Three of four patients had tocilizumab administration within 10 h of the onset of febrile neutropenia. In critically ill patients with COVID-19, Gordon et al. demonstrated a benefit of 90-day probability of survival in patients who were able to receive tocilizumab within 24 h after intensive care unit admission [32]. This finding was in line with our observation that tocilizumab could be more effective when administered early in the disease course because organ dysfunction was probably more reversible, but more randomized control studies are required to determine the optimal timing of tocilizumab administration.

Two of the patients with mortality were both female, while only two of five (40%) patients with successful recovery from severe sepsis/septic shock were female. Papathanassoglou et al. performed a systemic review on the influence of gender on outcomes in critically ill adult patients with sepsis [33]. They suggested that the impact of gender on sepsis outcomes was equivocal. Hatamabadi et al. retrospectively reviewed 357 adult patients with febrile neutropenia [34]. They found that gender was not significantly correlated with mortality. Recently, a prospective cohort study presented at the 2022 ASCO Breakthrough meeting demonstrated that there was no significant association with gender on the outcome of 131 children experiencing febrile neutropenia [35]. However, in general, there is limited literature in the field related to febrile neutropenia in children.

Although it seems that tocilizumab is helpful in suppressing the systemic inflammatory response in children with febrile neutropenia, some serious drawbacks may occur. Pawar et al. reported that the risk of bacterial, viral, and opportunistic infections was increased in patients with rheumatoid arthritis receiving tocilizumab [36]. Furthermore, tocilizumab may hypothetically induce encephalopathy because neurologic toxicity occurred shortly after tocilizumab administration for CRS following CAR T-cell therapy for leukemia [37]. Whether both effects are relevant in pediatric patients receiving tocilizumab for febrile neutropenia with severe sepsis/septic shock is unclear.

Some safety profiles of tocilizumab have been understood from the pooled data from five core phase 3 clinical trials for autoimmune diseases [38]. Common safety issues included serious infections, opportunistic infections, gastrointestinal perforations, malignancy, myocardial infarction, and stroke. However, there are some long-term questions requiring further investigation to answer clearly [39]. First, tocilizumab was well known to increase lipid levels. Some studies with a longer period of follow-up time did not show a trend for an increase in cardiovascular disease [40,41,42]. Second, infection rates in these long-term extension studies remained stationary [40,41,42]. Lin et al. recently evaluated the efficacy and safety of tocilizumab therapy in 114 patients with rheumatoid arthritis [43]. Isoniazid prophylaxis was given for 23 patients with old or latent tuberculosis infections, and pre-emptive antiviral treatment was given for 11 patients with chronic hepatitis B. None of them had a reactivation of tuberculosis and hepatitis B during the 3 years of follow-up. Their findings indicated that some infections could be prevented with an appropriate prophylactic or pre-emptive strategy when patients with underlying risk factors of infections underwent tocilizumab therapy. Unlike those reports for autoimmune disease in which tocilizumab was used for a much longer course and in adult patients, our treatment involved a single dose of tocilizumab in pediatric patients. Frigault MJ et al. compared 166 patients who received tocilizumab for grade 1 CRS after CAR-T therapy with 225 patients who did not. They found that 1–3 doses of tocilizumab for grade 1 CRS after CAR-T therapy was not associated with increased infection risk [44].

Our study has several limitations. First, this is a retrospective observational study with a small number of cases, making it difficult to clarify the relationship regarding the effectiveness of tocilizumab. Second, this is a single-center study with a small number of cases. The statistical power is too low to be convincing. Third, selection bias or confounding factors (e.g., gender, underlying health status, etc.) may be present in this study. All of the above could potentially impact the interpretation of our observational results. Therefore, a prospective study to enroll more patients is warranted to overcome our limitations.

In conclusion, Il-6 blockade with tocilizumab is a potential therapeutic strategy for severe sepsis/septic shock in children with febrile neutropenia. It resulted in clinical improvement and reduced mortality in this small case series of patients. Further research to investigate the efficacy and long-term safety in a larger number of patients with a longer follow-up is needed.

## Figures and Tables

**Table 1:** Clinical and laboratory features of seven febrile neutropenic patients with severe sepsis/septic shock.

	Case 1	Case 2	Case 3	Case 4	Case 5	Case 6	Case 7
Age (years) of the occurrence of FN	9.6	6.7	15.6	17.3	14.1	11.9	13.4
Sex	Female	Male	Female	Male	Female	Female	Male
Primary disease	AML	SAA	AML	MaGCT	ALL	SAA	SAA
Treatment before FN	Mitoxantrone, etoposide	Methylprednisolone, ATG, cyclosporine	Fludarabine, cytarabine	Gemcitabine, paclitaxal	Cyclosporine	Methylprednisolone, ATG, cyclosporine	Methylprednisolone, ATG, cyclosporine
Duration of neutropenia before FN	7 days	2 days	4 days	2 days	47 days	20 days	9 days
Antibiotic therapy before FN	Vancomycin, levofloxacin	No	Vancomycin, levofloxacin	No	Teicoplanin, meropenem	Teicoplanin, meropenem, amikacin	No
Antibiotic therapy after FN	Vancomycin, meropenem	Teicoplanin, meropenem	Teicoplanin, meropenem	Teicoplanin, meropenem	Teicoplanin, meropenem, amikacin	Teicoplanin, meropenem, amikacin	Teicoplanin, meropenem
Tocilizumab therapy	Yes	Yes	Yes	Yes	No	No	No
Time of tocilizumab therapy after FN	5 days	9 h	9.5 h	7 h	ND	ND	ND
Infectious pathogens of FN	Parainfluenza virus-3	None	*Brevundimonas* species	*Aeromonas* species	*Staphylococcus pettenkoferi*	*Ralstonia mannitolilytica*, *Stenotrophomonas maltophilia*	None
IL-6 (pg/mL)	1200	672	27,094	30,509	838	2191	ND
CRP (mg/L)	407.4	ND	26.75	178	65.34	243.41	136.01
PCT(ng/mL)	7.4	ND	ND	>88	0.84	8.87	19.29
Ferritin (ng/mL)	6890	ND	ND	14,891	47,327	1567	991
Circulatory failure	Absent	Present	Present	Present	Present	Present	Present
Organ dysfunction (other than circulatory failure)	Respiratory Neurological	No	Renal Hepatic	Respiratory Renal Hepatic	Respiratory Renal Hepatic	Respiratory	No
Severe sepsis	Yes	Yes	Yes	Yes	Yes	Yes	Yes
Septic shock	No	Yes	Yes	Yes	Yes	Yes	Yes
PICU admission	Yes	No	No	Yes	Yes	Yes	Yes
Mortality due to severe sepsis/septic shock	No	No	No	No	Yes	Yes	No

ALL: acute lymphoblastic leukemia; AML: acute myeloid leukemia, ATG: antithymocyte globulin; CRP: C-reactive protein; FN: febrile neutropenia; MaGCT: malignant germ cell tumor; ND: not done; PCT: procalcitonin; PICU: pediatric intensive care unit; SAA: severe aplastic anemia.

**Table 2:** Comparisons between patients who received tocilizumab or no tocilizumab.

	Tocilizumab (N = 4)	No Tocilizumab (N = 3)	*p*
IL-6 (pg/mL) (median, range)	14,147 (672–30,509)	1514.5 (838–2191)	0.8
CRP (mg/L) (median, range)	178 (26.75–407.4)	136.01 (65.34–243.41)	1
PCT (ng/mL) (median, range)	47.7 (7.4–88)	8.87 (0.84–19.29)	0.8
Ferritin (ng/mL) (median, range)	10,890.5 (6890–14,891)	1567 (997–47,327)	0.8
Duration of neutropenia before FN (days) (median, range)	3 (2–7)	20 (9–47)	0.049
PICU admission			0.43
Yes	2	3	
No	2	0	
Mortality			0.14
Yes	0	2	
No	4	1	

CRP: C-reactive protein; FN: febrile neutropenia; PCT: procalcitonin; PICU: pediatric intensive care unit.

## Data Availability

Data are contained within the article.

## References

[1] Davis K., Wilson S. (2020). Febrile neutropenia in paediatric oncology. Paediatr Child Health.

[2] Agulnik A. (2023). Management of septic shock in children with cancer-Common challenges and research priorities. J. Pediatr..

[3] Lehrnbecher T., Robinson P.D., Ammann R.A., Fisher B., Patel P., Phillips R., Beauchemin M.P., Carlesse F., Castagnola E., Davis B.L. (2023). Guideline for the Management of Fever and Neutropenia in Pediatric Patients with Cancer and Hematopoietic Cell Transplantation Recipients: 2023 Update. J. Clin. Oncol..

[4] de Castro R.E.V., Medeiros D.N.M., Prata-Barbosa A., de Magalhaes-Barbosa M.C. (2020). Surviving Sepsis Campaign International Guidelines for the Management of Septic Shock and Sepsis-Associated Organ Dysfunction in Children. Pediatr. Crit. Care Med..

[5] Evans L., Rhodes A., Alhazzani W., Antonelli M., Coopersmith C.M., French C., Machado F.R., McIntyre L., Ostermann M., Prescott H.C. (2021). Surviving Sepsis Campaign: International Guidelines for Management of Sepsis and Septic Shock 2021. Crit. Care Med..

[6] Weiss S.L., Peters M.J., Alhazzani W., Agus M.S.D., Flori H.R., Inwald D.P., Nadel S., Schlapbach L.J., Tasker R.C., Argent A.C. (2020). Surviving sepsis campaign international guidelines for the management of septic shock and sepsis-associated organ dysfunction in children. Intensive Care Med..

[7] Azevedo R.T., Araujo O.R., Petrilli A.S., Silva D.C.B. (2023). Children with malignancies and septic shock—An attempt to understand the risk factors. J. Pediatr..

[8] Diorio C., Shaw P.A., Pequignot E., Orlenko A., Chen F., Aplenc R., Barrett D.M., Bassiri H., Behrens E., DiNofia A.M. (2020). Diagnostic biomarkers to differentiate sepsis from cytokine release syndrome in critically ill children. Blood Adv..

[9] Huang L., Zhao X., Qi Y., Li H., Ye G., Liu Y., Zhang Y., Gou J. (2020). Sepsis-associated severe interleukin-6 storm in critical coronavirus disease 2019. Cell. Mol. Immunol..

[10] Shankar-Hari M., Vale C.L., Godolphin P.J., Fisher D., Higgins J.P.T., Spiga F., Savovic J., Tierney J., Baron G., WHO Rapid Evidence Appraisal for COVID-19 Therapies (REACT) Working Group (2021). Association Between Administration of IL-6 Antagonists and Mortality Among Patients Hospitalized for COVID-19: A Meta-analysis. JAMA J. Am. Med. Assoc..

[11] Wang B., Wang Q., Liang Z., Yin Y., Wang L., Wang Q., Li Y., Ou J., Ren H., Dong Y. (2023). Tocilizumab, an IL6-receptor antibody, proved effective as adjuvant therapy for cytokine storm induced by severe infection in patients with hematologic malignancy. Ann. Hematol..

[12] Goldstein B., Giroir B., Randolph A., International Consensus Conference on Pediatric S. (2005). International pediatric sepsis consensus conference: Definitions for sepsis and organ dysfunction in pediatrics. Pediatr. Crit. Care Med..

[13] Chen S.H., Yang C.P., Jaing T.H., Lai J.Y., Hung I.J. (2012). Catheter-related bloodstream infection with removal of catheter in pediatric oncology patients: A 10-year experience in Taiwan. Int. J. Clin. Oncol..

[14] Jaing T.H., Chang C.C., Chang T.Y., Chen S.H., Wen Y.C., Tsay P.K. (2020). Diagnostic Value of C-reactive Protein and Interleukin-8 in Risk Stratification of Febrile Neutropenic Children with Allogeneic Hematopoietic Stem Cell Transplantation. Sci. Rep..

[15] Azoulay E., Mokart D., Pene F., Lambert J., Kouatchet A., Mayaux J., Vincent F., Nyunga M., Bruneel F., Laisne L.M. (2013). Outcomes of critically ill patients with hematologic malignancies: Prospective multicenter data from France and Belgium—A groupe de recherche respiratoire en reanimation onco-hematologique study. J. Clin. Oncol..

[16] Soares M., Bozza F.A., Azevedo L.C., Silva U.V., Correa T.D., Colombari F., Torelly A.P., Varaschin P., Viana W.N., Knibel M.F. (2016). Effects of Organizational Characteristics on Outcomes and Resource Use in Patients With Cancer Admitted to Intensive Care Units. J. Clin. Oncol..

[17] Que Y., Hu C., Wan K., Hu P., Wang R., Luo J., Li T., Ping R., Hu Q., Sun Y. (2022). Cytokine release syndrome in COVID-19: A major mechanism of morbidity and mortality. Int. Rev. Immunol..

[18] Brown K.A., Brown G.A., Lewis S.M., Beale R., Treacher D.F. (2016). Targeting cytokines as a treatment for patients with sepsis: A lost cause or a strategy still worthy of pursuit?. Int. Immunopharmacol..

[19] Moore J.B., June C.H. (2020). Cytokine release syndrome in severe COVID-19. Science.

[20] de Araujo O.R., Salomao R., Brunialti M.K.C., da Silva D.C.B., Senerchia A.A., de Moraes Costa Carlesse F.A., Petrilli A.S. (2017). Cytokine Kinetics in Febrile Neutropenic Children: Insights on the Usefulness as Sepsis Biomarkers, Influence of Filgrastim, and Behavior of the IL-23/IL-17 Pathway. Mediat. Inflamm..

[21] Tapia L.I., Olivares M., Torres J.P., De la Maza V., Valenzuela R., Contardo V., Tordecilla J., Alvarez A.M., Varas M., Zubieta M. (2021). Cytokine and chemokine profiles in episodes of persistent high-risk febrile neutropenia in children with cancer. Cytokine.

[22] Song J., Park D.W., Moon S., Cho H.J., Park J.H., Seok H., Choi W.S. (2019). Diagnostic and prognostic value of interleukin-6, pentraxin 3, and procalcitonin levels among sepsis and septic shock patients: A prospective controlled study according to the Sepsis-3 definitions. BMC Infect. Dis..

[23] Zhang Y., Li B., Ning B. (2022). Evaluating IL-6 and IL-10 as rapid diagnostic tools for Gram-negative bacteria and as disease severity predictors in pediatric sepsis patients in the intensive care unit. Front. Immunol..

[24] Thompson D.K., Huffman K.M., Kraus W.E., Kraus V.B. (2012). Critical appraisal of four IL-6 immunoassays. PLoS ONE.

[25] Thao P.T.N., Tra T.T., Son N.T., Wada K. (2018). Reduction in the IL-6 level at 24 h after admission to the intensive care unit is a survival predictor for Vietnamese patients with sepsis and septic shock: A prospective study. BMC Emerg. Med..

[26] Morris E.C., Neelapu S.S., Giavridis T., Sadelain M. (2022). Cytokine release syndrome and associated neurotoxicity in cancer immunotherapy. Nat. Rev. Immunol..

[27] Nellan A., McCully C.M.L., Cruz Garcia R., Jayaprakash N., Widemann B.C., Lee D.W., Warren K.E. (2018). Improved CNS exposure to tocilizumab after cerebrospinal fluid compared to intravenous administration in rhesus macaques. Blood.

[28] Nishimoto N., Terao K., Mima T., Nakahara H., Takagi N., Kakehi T. (2008). Mechanisms and pathologic significances in increase in serum interleukin-6 (IL-6) and soluble IL-6 receptor after administration of an anti-IL-6 receptor antibody, tocilizumab, in patients with rheumatoid arthritis and Castleman disease. Blood.

[29] Muccioli L., Pensato U., Cani I., Guerra L., Provini F., Bordin G., Riccioli L.A., Lodi R., Tinuper P., Bisulli F. (2020). COVID-19-related encephalopathy presenting with aphasia resolving following tocilizumab treatment. J. Neuroimmunol..

[30] Hernandez-Parra H., Reyes-Hernandez O.D., Figueroa-Gonzalez G., Gonzalez-Del Carmen M., Gonzalez-Torres M., Pena-Corona S.I., Floran B., Cortes H., Leyva-Gomez G. (2023). Alteration of the blood-brain barrier by COVID-19 and its implication in the permeation of drugs into the brain. Front. Cell. Neurosci..

[31] Sekino N., Selim M., Shehadah A. (2022). Sepsis-associated brain injury: Underlying mechanisms and potential therapeutic strategies for acute and long-term cognitive impairments. J. Neuroinflamm..

[32] Investigators R.-C., Gordon A.C., Mouncey P.R., Al-Beidh F., Rowan K.M., Nichol A.D., Arabi Y.M., Annane D., Beane A., van Bentum-Puijk W. (2021). Interleukin-6 Receptor Antagonists in Critically Ill Patients with Covid-19. N. Engl. J. Med..

[33] Papathanassoglou E., Middleton N., Benbenishty J., Williams G., Christofi M.D., Hegadoren K. (2017). Systematic review of gender- dependent outcomes in sepsis. Nurs. Crit. Care.

[34] Hatamabadi H., Arhami Dolatabadi A., Akhavan A., Safari S. (2019). Clinical Characteristics and Associated Factors of Mortality in Febrile Neutropenia Patients; a Cross Sectional Study. Arch. Acad. Emerg Med..

[35] Dabas A., Sharma V., Singh A., Kriplani K., Gaind R., Gera R. (2023). Factors associated with adverse outcome in pediatric oncology patients with febrile neutropenia: A prospective cohort study from India. JCO Glob. Oncol..

[36] Pawar A., Desai R.J., Solomon D.H., Santiago Ortiz A.J., Gale S., Bao M., Sarsour K., Schneeweiss S., Kim S.C. (2019). Risk of serious infections in tocilizumab versus other biologic drugs in patients with rheumatoid arthritis: A multidatabase cohort study. Ann. Rheum. Dis..

[37] Brudno J.N., Kochenderfer J.N. (2019). Recent advances in CAR T-cell toxicity: Mechanisms, manifestations and management. Blood Rev..

[38] Schiff M.H., Kremer J.M., Jahreis A., Vernon E., Isaacs J.D., van Vollenhoven R.F. (2011). Integrated safety in tocilizumab clinical trials. Arthritis Res. Ther..

[39] Jones G., Panova E. (2018). New insights and long-term safety of tocilizumab in rheumatoid arthritis. Ther. Adv. Musculoskelet. Dis..

[40] Nishimoto N., Miyasaka N., Yamamoto K., Kawai S., Takeuchi T., Azuma J. (2009). Long-term safety and efficacy of tocilizumab, an anti-IL-6 receptor monoclonal antibody, in monotherapy, in patients with rheumatoid arthritis (the STREAM study): Evidence of safety and efficacy in a 5-year extension study. Ann. Rheum. Dis..

[41] Genovese M.C., Rubbert-Roth A., Smolen J.S., Kremer J., Khraishi M., Gomez-Reino J., Sebba A., Pilson R., Williams S., Van Vollenhoven R. (2013). Longterm safety and efficacy of tocilizumab in patients with rheumatoid arthritis: A cumulative analysis of up to 4.6 years of exposure. J. Rheumatol..

[42] Jones G., Wallace T., McIntosh M.J., Brockwell L., Gomez-Reino J.J., Sebba A. (2017). Five-year Efficacy and Safety of Tocilizumab Monotherapy in Patients with Rheumatoid Arthritis Who Were Methotrexate- and Biologic-naive or Free of Methotrexate for 6 Months: The AMBITION Study. J. Rheumatol..

[43] Lin C.T., Huang W.N., Hsieh C.W., Chen Y.M., Chen D.Y., Hsieh T.Y., Chen Y.H. (2019). Safety and effectiveness of tocilizumab in treating patients with rheumatoid arthritis—A three-year study in Taiwan. J. Microbiol. Immunol. Infect..

[44] Frigault M.J., Nikiforow S., Mansour M.K., Hu Z.H., Horowitz M.M., Riches M.L., Hematti P., Turtle C.J., Zhang M.J., Perales M.A. (2020). Tocilizumab not associated with increased infection risk after CAR T-cell therapy: Implications for COVID-19?. Blood.

